# Gliomasphere marker combinatorics: multidimensional flow cytometry detects CD44+/CD133+/ITGA6+/CD36+ signature

**DOI:** 10.1111/jcmm.13927

**Published:** 2018-11-22

**Authors:** Friedrich Erhart, Bernadette Blauensteiner, Gabriel Zirkovits, Dieter Printz, Klara Soukup, Simone Klingenbrunner, Katrin Fischhuber, René Reitermaier, Angela Halfmann, Daniela Lötsch, Sabine Spiegl‐Kreinecker, Walter Berger, Carmen Visus, Alexander Dohnal

**Affiliations:** ^1^ Department of Neurosurgery Medical University of Vienna Vienna Austria; ^2^ Institute of Neurology Medical University of Vienna Vienna Austria; ^3^ Department of Tumor Immunology St. Anna Kinderkrebsforschung Children's Cancer Research Institute Vienna Austria; ^4^ FACS Core Unit St. Anna Kinderkrebsforschung Children's Cancer Research Institute Vienna Austria; ^5^ Activartis Biotech GmbH Vienna Austria; ^6^ Institute for Cancer Research Comprehensive Cancer Center Medical University of Vienna Vienna Austria; ^7^ University Clinic for Neurosurgery Kepler University Hospital Johannes Kepler University Linz Austria

**Keywords:** flow cytometry, glioblastoma, gliomaspheres, molecular signature, multi‐color staining, stem‐like cell, viSNE

## Abstract

Glioblastoma is the most dangerous brain cancer. One reason for glioblastoma's aggressiveness are glioblastoma stem‐like cells. To target them, a number of markers have been proposed (CD133, CD44, CD15, A2B5, CD36, CXCR4, IL6R, L1CAM, and ITGA6). A comprehensive study of co‐expression patterns of them has, however, not been performed so far. Here, we mapped the multidimensional co‐expression profile of these stemness‐associated molecules. Gliomaspheres – an established model of glioblastoma stem‐like cells – were used. Seven different gliomasphere systems were subjected to multicolor flow cytometry measuring the nine markers CD133, CD44, CD15, A2B5, CD36, CXCR4, IL6R, L1CAM, and ITGA6 all simultaneously based on a novel 9‐marker multicolor panel developed for this study. The viSNE dimensionality reduction algorithm was applied for analysis. All gliomaspheres were found to express at least five different glioblastoma stem‐like cell markers. Multi‐dimensional analysis showed that all studied gliomaspheres consistently harbored a cell population positive for the molecular signature CD44+/CD133+/ITGA6+/CD36+. Glioblastoma patients with an enrichment of this combination had a significantly worse survival outcome when analyzing the two largest available The Cancer Genome Atlas datasets (MIT/Harvard Affymetrix: *P* = 0.0015, University of North Carolina Agilent: *P* = 0.0322). In sum, we detected a previously unknown marker combination – demonstrating feasibility, usefulness, and importance of high‐dimensional gliomasphere marker combinatorics.

## 1 INTRODUCTION

Glioblastoma is the most aggressive and most frequent malignant brain tumor in adults.^1^ Classical treatment comprising surgery, radiotherapy, and chemotherapy is only temporarily effective. The median survival in adult patients is less than 2 years.

One reason for glioblastoma's resistance to a number of therapies is the presence of stem‐like glioblastoma cells.^2^ Despite initial hesitation in the scientific community towards a concept of stem‐like cells in glioblastoma, they are now mainly accepted as existent.^3^, ^4^, ^5^, ^6^, ^7^ These stem‐like cells share a transcriptional profile with neural stem cells.^8^ Similar to how neural stem cells grow as neurospheres*in vitro*, glioblastoma stem‐like cells grow as “gliomaspheres”*in vitro*. These gliomaspheres resemble the original tumor more than traditional adherent cell culture of glioblastoma cells does.^9^

Glioblastoma stem‐like cells have first been described by Singh et al in 2004.^10^ To prove stemness of stem‐like cells, xenotransplantation experiments were performed showing that as little as 100 cells could initiate tumor formation in NOD‐SCID mouse brains. To identify stem‐like cells, Singh et al used the surface marker CD133. Since then, CD133 has become the most studied marker for glioblastoma stem‐like cells. It has been connected to radioresistance.^2^ It has been shown to be essential for stem cell maintenance.^11^ It has been visualised in positron emission tomography to monitor stem‐like glioblastoma cells.^12^ And it has been connected spatially and functionally to perivascular stem cell niches^13^, ^14^ or hypoxic niches.^15^ There was, however, also controversy regarding its suitability as a marker to unambiguously identify glioblastoma stem‐like cells.^16^

Therefore, also other potential markers have been investigated and discussed. An association with stemness has been established for a number of them in glioblastoma: A2B5, a glial progenitor cell marker, has been shown to identify glioblastoma cells with stem‐like properties.^17^, ^18^ L1CAM is evidently necessary for the maintenance and survival of glioblastoma stem‐like cells *in vivo* and*in vitro*.^19^ CXCR4 is overexpressed in glioblastoma stem‐like cells as opposed to bulk glioblastoma cells^20^ and its inhibition reduces the self‐renewal capacity of glioblastoma stem‐like cells.^21^ CD15 (SSEA‐1, Lewis X) has been shown to be an enrichment factor for glioblastoma stem‐like cells.^22^ IL6R (Interleukin‐6 receptor) is preferentially expressed on glioblastoma stem‐like cells and targeting it via eg short hairpin RNAs (shRNAs) reduces gliomasphere formation capacity.^23^ CD44 has been implicated with the perivascular stem cell niche and a cell population enriched with glioblastoma stem‐like cells.^24^ Also, CD44 signaling can enhance glioblastoma stem‐like cell phenotypes.^25^ ITGA6 (Integrin alpha‐6) has been identified as a glioblastoma stem‐like cell enrichment marker and a potential therapeutic target.^26^ Apart from that, ITGA6 inhibition is necessary for some forms of glioblastoma stemness suppression.^27^ CD36 is specific for glioblastoma stem‐like cells and drives glioblastoma progression.^28^

All in all, nine different surface molecules (CD133, A2B5, L1CAM, CXCR4, CD15, IL6R, CD44, ITGA6 and CD36) have repeatedly been associated with glioblastoma stem‐like cells in the literature. These nine molecules are consistently summarised as a particularly relevant set of stemness‐related markers in glioblastoma – eg by Sundar et al and by Brescia et al.^29^, ^30^, ^31^, ^32^, ^33^ That is why we chose to focus on these nine markers.

While individual marker expression has been extensively studied (see above), their overall co‐expression motifs are largely unknown so far. Various papers address double positive expression patterns in glioblastoma cells, eg for CD133+/IL6R+ cells^23^ or A2B5+/CD133+^18^ cells. But multidimensional stemness marker combination studies are still rare in glioblastoma. One such investigation is that by Stuelten et al who evaluated the expression patterns of seven markers generally associated with stemness (CD15, CD24, CD44, CD133, CD166, CD326, PgP) in the NCI60 tumor cell line panel that comprises lung, colon, breast, skin cancer as well as hematopoietic malignancies, and brain cancer.^34^ That study, however, did not focus on stemness markers specific for glioblastoma but rather used a generic stemness marker panel. To our knowledge, there is so far no comprehensive study on the nine stemness markers specifically identified in glioblastoma and their respective co‐expression. To date, this represents a relevant gap in the neuroscientific landscape of glioblastoma stemness markers and how they are interrelated.

The objective of our research efforts was thus to address the present gap via introducing high‐dimensional measurement techniques and to establish co‐expression patterns of these 9 relevant glioblastoma stemness markers (Figure S1). We decided to approach the goal via the*in vitro*‐study of gliomaspheres combined with a multicolor flow cytometry antibody panel comprising all nine glioblastoma stem‐like cell molecules at once. To control for biological variation, we chose to use seven gliomasphere systems from three different sources: the stem‐like cell lines NCH421K and NCH644 that already grow in gliomaspheres,^35^, ^36^ gliomaspheres derived from the classical glioblastoma cell lines U87MG and U251MG^37^ and gliomaspheres derived from patient material (Linz1, Linz2, Gli16) gained via surgery during a clinical trial (J. Buchroithner, F. Erhart, J. Pichler, G. Widhalm, M. Preusser, G. Stockhammer, M. Nowosielski, S. Iglseder, C.F. Freyschlag, S. Oberndorfer, K. Bordihn, G. von Campe, M. Hoffermann, R. Ruckser, K. Rössler, F. Erhart, S. Spiegl‐Kreinecker, M.B. Fischer, T. Czech, C. Visus, G. Krumpl, T. Felzmann & C. Marosi, data currently being submitted).

To integrate the 9‐dimensional flow cytometry data, we used the viSNE algorithm^38^ that condenses multi‐dimensional information to two dimensions, which can then be visualised.

The question of a potential clinical importance of observed marker combinations was subsequently studied using The Cancer Genome Atlas (TCGA) datasets. Also, we illustrated the biological pathways typical for the most common stem‐like cell marker combination via eg the Database for Annotation, Visualization and Integrated Discovery (DAVID) bioinformatics system.

As a result, we successfully mapped the hitherto unknown co‐expression combinatorics of 9 stemness‐associated markers. This led to the discovery of a novel marker combination (CD44+/CD133+/ITGA6+/CD36+) found consistently on all studied model systems – proving the practicability, utility and relevance of multidimensional approaches for studying gliomaspheres.

## 2 MATERIALS AND METHODS

### 2.1 Glioblastoma cell source

The cell lines, U87MG (HTB‐14TM), and U251MG were obtained from the American Type Culture Collection (ATCC^®^, Manassas, VA, USA). The glioblastoma stem‐like cell lines NCH421K and NCH644^36^ were purchased from Cell Line Services (CLS, Eppelheim, Germany). Linz1, Linz2, and Gli16 were derived from glioblastoma patients from a phase II clinical trial (NCT01213407) via surgery, dissociation, and subsequent*in vitro* culture (J. Buchroithner, F. Erhart, J. Pichler, G. Widhalm, M. Preusser, G. Stockhammer, M. Nowosielski, S. Iglseder, C.F. Freyschlag, S. Oberndorfer, K. Bordihn, G. von Campe, M. Hoffermann, R. Ruckser, K. Rössler, F. Erhart, S. Spiegl‐Kreinecker, M.B. Fischer, T. Czech, C. Visus, G. Krumpl, T. Felzmann & C. Marosi, data currently being submitted). All patients had given their written informed consent that their cell material can be processed for further studies in addition to the clinical trial. The ethics committee of the federal state of Upper Austria approved the research (approval number TRX 2/P‐II‐018).

### 2.2 Gliomasphere culture

Gliomaspheres were generated in analogy to well established standard protocols.^37^, ^39^, ^40^, ^41^ Briefly, glioblastoma cells were transferred from T75 to T25 flasks (to facilitate cell‐cell contacts) with serum‐free media supplemented with growth factors – as typically used for a spheric phenotype^15^, ^36^, ^42^: DMEM/Nutrient Mixture F‐12 medium (DMEM, Gibco, Life Technologies, Paisley, UK) supplemented with 20% BIT‐serum free supplement (bovine serum albumin, insulin, transferrin), human recombinant epidermal growth factor and human basic fibroblast growth factor at 20 ng/mL each (all STEMCELL Technologies, Vancouver, BC, Canada). For passaging and plating, spheres were transferred into conical tubes, centrifuged (200 *g*, 5 minutes), resuspended in serum‐free medium, dissociated into single‐cell suspensions using trituration (60‐70 times using a Pasteur pipette) and re‐plated. BIT as well as growth factors were added freshly to maintain the concentration after each passage. Tumor spheres were observed under a light microscope every day.

### 2.3 Flow cytometric analysis

#### 2.3.1 Cell count

The expression of stem cell markers was assessed by flow cytometry. Cells were harvested and counted in a volume of 100 μL on a BD LSR II flow cytometer (Becton Dickinson, San Diego, CA, USA) using TruCountTM tubes (BD Biosciences, San José, CA, USA). The absolute count of the cell population was obtained using FlowJo Analysis Software V10 (Tree Star, Ashland, OR, USA).

#### 2.3.2 Instrumentation

A flow cytometer (LSR II; BD Biosciences) equipped with Diva V8.0 software was used for 9‐color phenotypic analysis. Laser alignment (405‐nm violet laser, 488‐nm blue laser, 640‐nm red laser) was verified with beads prior to running tumor cell samples. The nine colors used in the analysis were compensated twice to account for spectral overlap emitted by the fluorochromes within each laser as well as across the lasers using a semi‐automated as well as a manual process by the application of compensation beads (BD Biosciences) coated with anti‐human Ig antibodies. The beads were incubated with each individual antibody used in the nine‐color panel for 15 minutes at room temperature. Unstained and stained beads were run individually, 5000 events were recorded, and data were imported into Diva V8.0 software including compensation matrices for automatic as well as additional manual compensation.

#### 2.3.3 9‐color tumor cell labeling

For the evaluation of the stem cell phenotype of the gliomaspheres, >20 × 10^5^ disaggregated sphere cells were stained with a stem cell‐specific antibody panel and analysed on a BDTM LSR II flow cytometer. To a volume of 100 μL cell suspension, 5 μL antibody mix was added, mixed and incubated for 20 minutes at 4°C in the dark and washed twice with 250 μL DPBS or FACS buffer. For the full set of antibodies see Table S1. 7‐AAD was used as viability dye. Additionally, unstained controls as well as appropriate human isotype controls were added to the panel according to the manufacturer's recommendations.

#### 2.3.4 Data interpretation and definition of “positive” markers

Cytometer measurements were analysed using FlowJo V10. A difference of at least 10% in the mean fluorescence intensity (MFI) values between epitope‐specific antibodies and isotype antibodies in combination with a visually distinguishable shift of the histogram was considered a “positive” staining.

### 2.4 viSNE analysis

The viSNE dimensionality reduction tool was used to visualise multidimensional surface molecule expression patterns on a single cell basis. Data were analysed in MatLab (MathWorks, Natick, MA, USA) to create a viSNE map that relies on minimizing differences between high‐ and low‐dimensional spaces and produces a 2‐dimensional plot. Briefly, a pairwise distance matrix is calculated in high dimensional space, which is transformed to a similarity matrix using a Gaussian kernel. The points are randomly mapped in low‐dimensional space and iteratively rearranged to minimise the divergence between high‐ and low‐dimensional similarity matrices.

### 2.5 TCGA analysis of GBM patient cohorts

In order to estimate the clinical relevance of our stem‐like cell signature (consensus signature of stemness markers expressed by all cell lines) GBM patient data was extracted from the open source TCGA cancer genome atlas to conduct Kaplan–Meier survival analyses. In total, data from two different sources was analysed, ie the Affymetrix HT Human Genome U133a microarray (n = 539) by the Broad Institute of MIT and Harvard University and the AgilentG4502A_07_2 microarray analysis (n = 483) by the University of North Carolina. These are the two largest glioblastoma gene expression datasets available in the TCGA database. The respective third of the lowest and highest expression group of each patient cohort was compared after correction for data entries with missing data.

### 2.6 Marker combination pathway analysis

For the study of biological pathways relevant for stem‐like cell marker combinations, we worked with the DAVID as well as the Pathway Commons Network Visualizer (PCViz). Both were used according to the instructions provided.^43^, ^44^

### 2.7 Statistical analyses

The log‐rank test was used for comparison of survival curves. *P* < 0.05 were considered significant. All statistical analyses were carried out with the GraphPad Prism V5 software for Windows (GraphPad, San Diego, CA, USA).

## 3 RESULTS

### 3.1 Single‐color staining of nine molecules: All 7 gliomaspheres examined express at least 5 stemness‐associated markers

**Figure 1:**
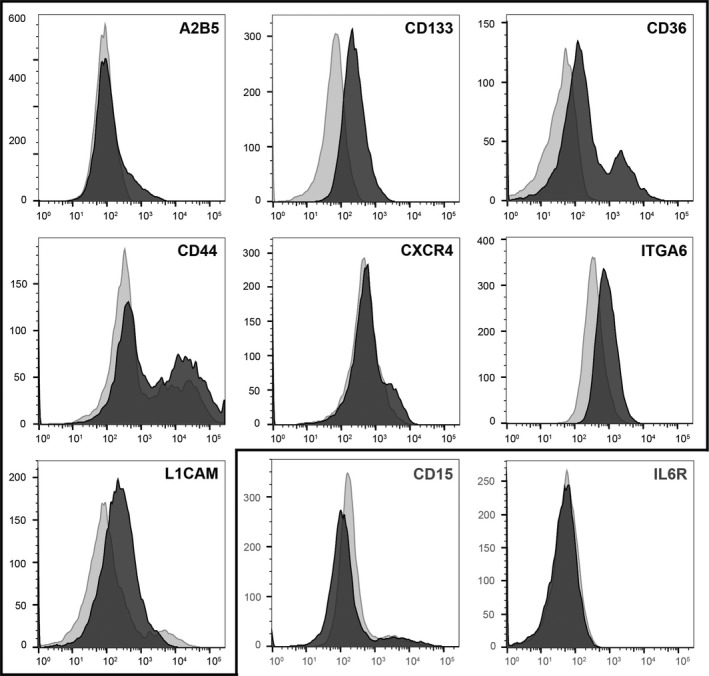
Flow cytometry histograms of NCH644 cells showing surface marker expression of 9 stemness‐associated molecules: A2B5, CD133, CD15, CD36, CD44, CXCR4, IL6R, ITGA6, and L1CAM. Framed histograms highlight positively expressed markers, while the other graphs indicate molecules that were not expressed by NCH644 cells. Dark‐gray curves represent expression levels of target‐specific antibodies and light‐gray curves isotypes (or for CD36 the unstained control as no isotype was available). *x* axis: marker expression, *y* axis: cell count. Note: the positivity of A2B5 and CXCR4 is better visible in the multicolor staining of Figure 2

As a first step, we evaluated the single‐color marker expression of the nine stemness‐associated molecules on all seven gliomaspheres. Positivity of a marker was defined as an increase in MFI of at least 10% compared with the isotype antibody (or unstained cells in the case of CD36) plus a visually identifiable shift of the histogram. Both criteria had to be met. Figure 1 depicts the single‐marker analysis of NCH644 gliomaspheres as an example. Single‐color analysis of cells stained with epitope‐specific antibodies (vs cells stained with isotype antibodies) indicates that all seven gliomaspheres have the capacity to express at least five stemness‐associated markers (Table 1 and Table S2). NCH421K and NCH644 gliomaspheres showed the highest number of markers as they were positive for eight surface molecules (A2B5, CD15, CD44, CD133, CXCR4, ITGA6, L1CAM, CD36) and only negative for IL6R. The gliomaspheres expressing the lowest number of just five molecules were Linz1 and Gli16. Linz2, U87MG and U251MG expressed 6 different stemness‐associated molecules. Of the nine molecules evaluated, eight were found on at least one gliomasphere model. IL6R was the only molecule not present on any of the seven gliomasphere models.

**Table 1:** Evaluation of single marker expression of the 7 cell lines. Stemness‐associated surface molecules (A2B5, CD133, CD15, CD36, CD44, CXCR4, IL6R, ITGA6, L1CAM) are shown horizontally, cell lines (Linz1, Linz2, Gli16, U87MG, U251MG, NCH421K, NCH644) vertically. Numbers indicate technical replicates, i.e. how often markers could be determined in two independent experiments (cell line cultivation and flow cytometry). Dark grey‐tinted areas equal two and light grey‐tinted areas one successful marker identification. Empty spots indicate no measurable expression. The exact MFI values are given in Table S2

		Single staining markers								
		A2B5	**CD133**	CD15	**CD36**	**CD44**	CXCR4	IL6R	**ITGA6**	L1CAM
Cell lines	Linz1		2		2	2	2		2	
	Linz2	2	2		2	2	2		1	
	Gli16	2	2		2	2			2	
	U87MG	1	2		2	2	1		2	
	U251MG	1	2	1	2	2			2	
	NCH421K	2	2	2	2	2	2		2	1
	NCH644	2	2	1	2	2	2		2	2

### 3.2 Single‐color staining of nine molecules: The most frequent set of markers present is CD44, CD133, ITGA6 and CD36

When looking at the most abundant combination of factors in this single‐color analysis, we found that the set of markers expressed most often was CD44, CD133, ITGA6 and CD36. Of the seven gliomasphere models, this combination could be observed in all of them in at least one measurement (Table 1). Hence, all gliomasphere models shared the same minimal combination of markers.

As single‐color flow cytometry does not allow conclusions about the co‐expression of specific markers by a specific subset of cells, we proceeded to perform a multi‐color staining to address this issue.

### 3.3 Multi‐color staining of nine molecules: All possible double‐marker combinations are detectable

As the first step of the multi‐color analysis, we investigated the existence of double‐positive populations. As depicted in Table S3, all theoretical double‐marker combinations of the eight measurable markers (except for IL6R) could be found in at least one of the seven gliomasphere systems. Overall, of the 232 possible gliomasphere‐specific combinations based on the previous single‐color measurement (Table S3), only 16 (6.9%) were not detectable.

### 3.4 Multi‐color staining of nine molecules: The core signature CD44+/CD133+/ITGA6+/CD36+ is present in all 7 gliomaspheres

**Figure 2:**
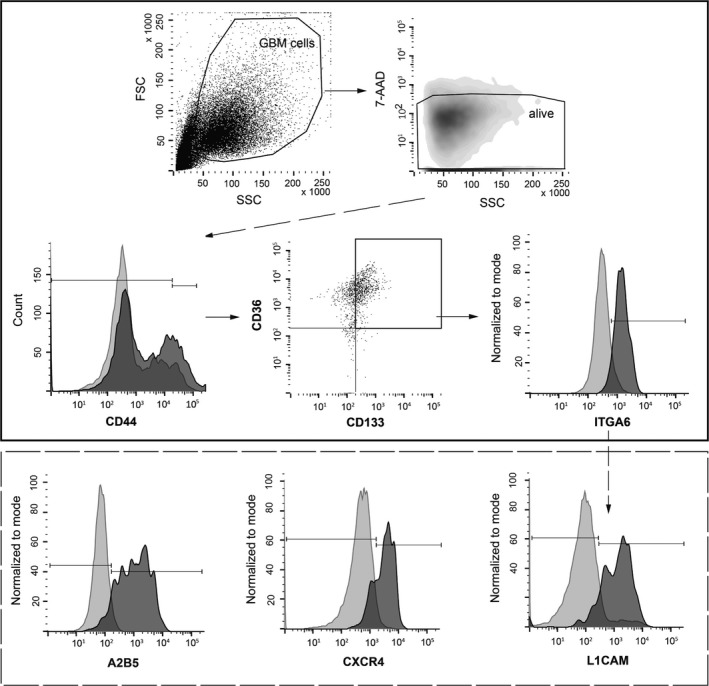
The gating strategy for gliomasphere characterization demonstrated on NCH644. In a first step, living cells were gated, identified by 7‐AAD negative cells and side scatter (SSC) properties. Next, CD44+ gated cells were analysed for a CD133+ and CD36+ cell population. Finally, ITGA6+ cells were analysed within this cell subset. 4‐marker positive cells, CD44+/CD133+/CD36+/ITGA6+, were back‐gated to further identify additional markers

As a next step, we looked at the multidimensional multicolor data going beyond double‐positive combinations. We discovered that the above described combination of markers seen in the single‐color staining (CD44, CD133, ITGA6 and CD36) could also be detected as a co‐expression motif on a subgroup of cells that simultaneously express all four molecules. These CD44+/CD133+/ITGA6+/CD36+ cells were identified through the gating strategy of first selecting CD44+ cells, then gating on CD133/CD36+ cells and finally selecting ITGA6+ cells. Figure 2 depicts NCH644 gliomaspheres as an example for the gating strategy we applied.

The existence of this 4‐marker positive subgroup could be confirmed in all seven gliomaspheres. Hence, CD44+/CD133+/ITGA6+/CD36+ is the core signature that is consistently present in all seven gliomasphere systems as the common consensus multi‐marker combination – irrespective of the origin of the gliomasphere (stem‐like cell line, traditional glioblastoma cell line or patient‐derived). Interestingly, while all 7 gliomasphere models showed this core sub‐population, additionally, in five of them the core population was also positive for at least one further marker (Table 2). The exact gating process for all further gliomasphere models apart from NCH644 is depicted in Figures S2‐S7.

**Table 2:** Multi‐color staining results of the 9 stem cell markers in the 7 gliomasperes. In all 7 gliomaspheres, we could find a cellular population positive for the core signature CD44/CD133/CD36/ITGA6. In selected gliomaspheres, the cells from the core signature were in addition also positive for further markers

	Multi‐color staining markers				
Linz1	Core				
Linz2	Core				
Gli16	Core	+ A2B5			
U87MG	Core	+ A2B5			
U251MG	Core	+ A2B5			
NCH421K	Core	+ A2B5	+ CXCR4		+ CD15
NCH644	Core	+ A2B5	+ CXCR4	+ L1CAM	

### 3.5 Multi‐color staining of nine molecules: Integrated multidimensional analysis via the viSNE algorithm confirms the CD44+/CD133+/ITGA6+/CD36+ signature in all 7 gliomaspheres

**Figure 3:**
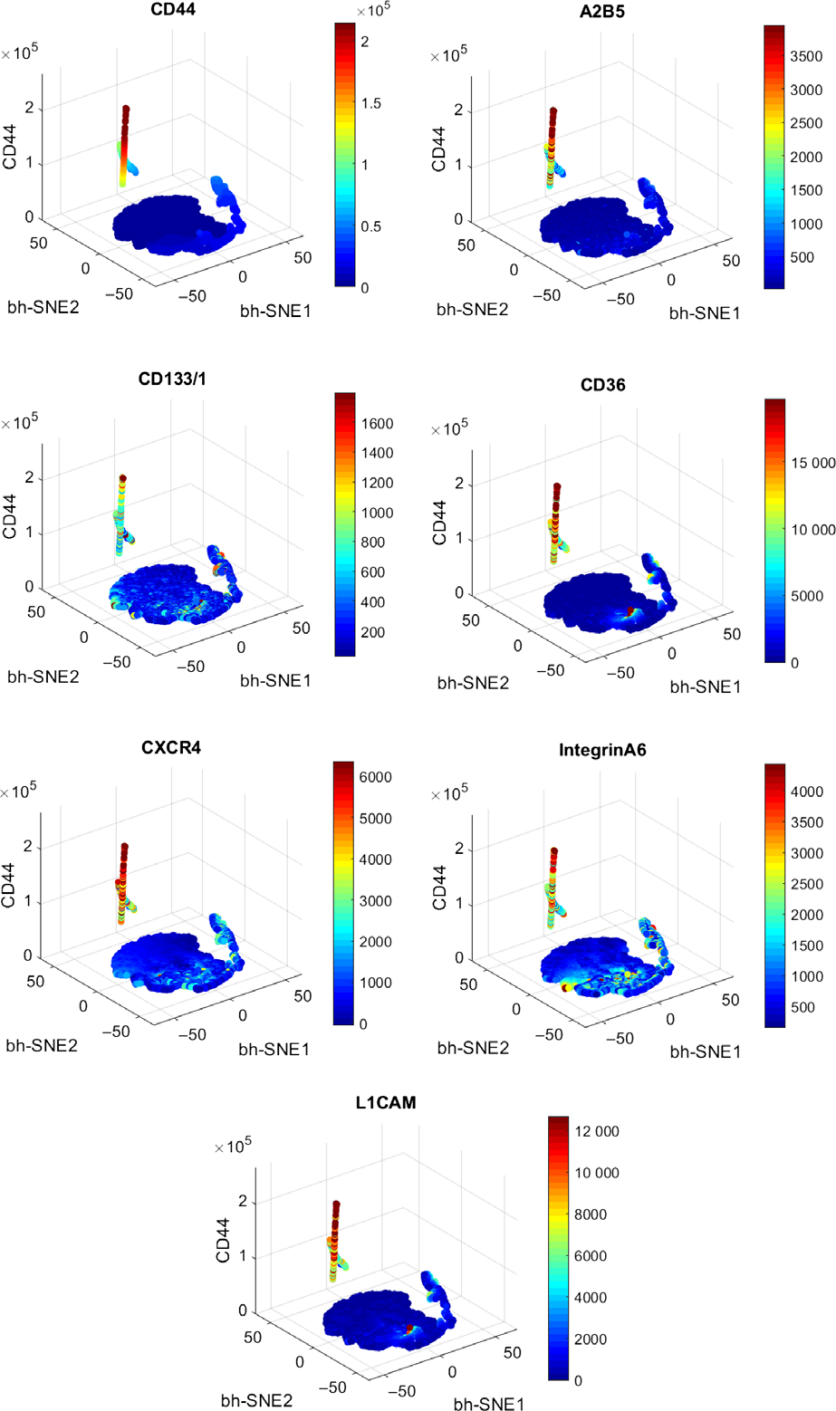
Results of the viSNE multidimensional algorithm analysis as shown for NCH644. The viSNE algorithm reduces multiple dimensions to two dimensions (“bh‐SNE1” and “bh‐SNE2”) that indicate the overall similarity of cells. The graphs show as a third (*z*‐)axis the expression of CD44. The color coding indicates the intensity of further markers and which cellular population is enriched. The algorithm clearly identifies the core stem‐like cell population as a distinct population

To integrate the 9‐dimensional marker information measured through multi‐color staining also by automatic analysis, we used the viSNE algorithm. It reduces multiple dimensions to two dimensions of “similarity.” Single markers can then be depicted as a third axis and a fourth marker can be visualised through color coding of intensity (shown for NCH644 in Figure 3). Across all of the seven gliomaspheres examined we could confirm the existence of the previously identified core signature (data not shown). We consistently found a population of cells with high‐intensity color coding of the markers CD44, CD133, CD36, and ITGA6 (shown for NCH644 in Figure 3 as an example).

### 3.6 TCGA survival data: The CD44+/CD133+/ITGA6+/CD36+ signature identified is associated with a significantly shorter survival

**Figure 4:**
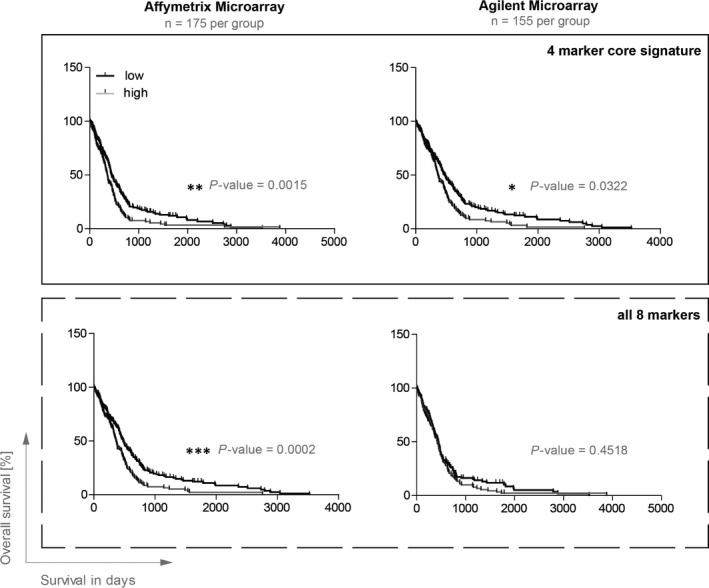
Core signature expression significantly correlates with a poor patient survival. A total of 2 different sized datasets, the MIT/Harvard (Affymetrix) cohort and the University of North Carolina (Agilent) cohort, were extracted from the open source The Cancer Genome Atlas and analysed for the core signature expression CD133+/CD36+/CD44+/ITGA6+ or all 9 markers (less the ganglioside A2B5 that is not measured in gene expression datasets). ***P < 0.001, **P < 0.01, *P < 0.05

Next, to assess the potential clinical importance of the core 4‐marker signature identified (CD44, CD133, ITGA6 and CD36), we analysed publicly available glioblastoma gene expression and survival datasets from the TCGA. We chose the MIT/Harvard University dataset (Affymetrix) and the University of North Carolina dataset (Agilent) as these two are the largest cohorts in the TCGA database. When splitting patients into groups with a “high” vs “low” expression profile of the 4‐marker signature (see Section 2), we could observe that patients with a “high” abundance of the four molecules lived significantly shorter in both datasets (Figure 4, MIT/Harvard: *P* = 0.0015, North Carolina: *P* = 0.0322). When combining all nine possible markers into a signature (excluding A2B5 as it is a ganglioside), an effect on survival was only visible in the MIT/Harvard dataset (*P* = 0.0002) but not in the North Carolina dataset (*P* = 0.4518).

### 3.7 In silico pathway analysis: Cell adhesion is among the biological processes connected to the CD44+/CD133+/ITGA6+/CD36+ signature

Then, we investigated the potential biological meaning of the identified marker combination. The DAVID analysis indicated that “cell adhesion,” “cell‐matrix adhesion,” and “ECM‐receptor interaction” are among the biological processes typical for the core combination (Table 3).

**Table 3:** Integrated pathway analysis of the core signature or the full signature. Results of the DAVID analysis of pathways that are enriched for the proteins of either the core signature (A) or the full set of stem‐like cell markers from the literature (B). Fold enrichment refers to the relative enrichment of the respective marker combination in the pathway

	Protein count	Fold enrichment	*P*‐value
(A) Core signature: CD44+CD133+CD36+ITGA6			
KEGG pathway			
Hematopoietic cell lineage	3	61	4.5E‐4
ECM‐receptor interaction	3	60	4.7E‐4
Gene ontology biological process			
Cell adhesion	3	27	2.2E‐3
Positive regulation of peptidyl‐tyrosine phosphorylation	2	102	1.5E‐2
Cell‐matrix adhesion	2	93	1.6E‐2
(B) Full set of markers: CD44+CD133+CD36+ITGA6+L1CAM+CXCR4+CD15+IL6R			
KEGG pathway			
Hematopoietic cell lineage	4	40	6.1E‐5
ECM‐receptor interaction	3	30	3.2E‐3
Gene ontology biological process			
Positive regulation of peptidyl‐tyrosine phosphorylation	3	77	4.9E‐4
Cell adhesion	4	18	6.5E‐4
Leukocyte migration	3	52	1.1E‐3

Also, we studied interaction pathways with the help of the PCViz that summarises findings from the literature. When entering the four signature genes, focusing on state change control interactions and zooming in on the 200 most relevant genes, we arrived at the interactome depicted in Figure S8. Apparently, Protein kinase C α (PRKCA) is a molecule that interacts with ITGA6, CD36 und CD44, while CD133 is not linked to PRKCA.

### 3.8 TCGA data: CD44+/CD133+/ITGA6+/CD36+ signature and relation to glioblastoma subtypes

Finally, we investigated the relation of the identified CD44+/CD133+/ITGA6+/CD36+ signature to the four glioblastoma molecular subtypes (mesenchymal, neural, proneural, and classical) as well as the isocitrate dehydrogenase 1 (IDH1) status. Again, TCGA expression data was used. Within the molecular subtypes, the proneural subtype had a significantly lower relative intensity of the CD44+/CD133+/ITGA6+/CD36+ signature than other subtypes in both datasets (MIT/Harvard: vs classical *P* = 0.031, vs mesenchymal subtype *P* = 0.006; North Carolina: vs neural: *P* = 0.005, vs mesenchymal: *P* = 0.032; data not shown).

When examining the relation to IDH1 status, we registered that the CD44+/CD133+/ITGA6+/CD36+ signature was significantly higher in IDH1 wild‐type patients than in IDH1‐mutated patients (*P* < 0.001) – in both datasets (see Figure S9).

## 4 DISCUSSION

### 4.1 Summary of key findings

With the work presented here, we describe gliomasphere marker co‐expression patterns and a novel, 4‐ marker molecule combination (CD44+/CD133+/ITGA6+/CD36+) that is consistently present in a cellular population of the seven different gliomasphere models. The signature is detectable by two different flow cytometry interpretation strategies: classical manual gating as well as automated multi‐dimensional population detection. TCGA data hints at a potential clinical relevance of the combination.

The main overall finding is that feasibility, utility and relevance of a multidimensional approach to gliomasphere research are shown here.

### 4.2 Limitations of the study

This study focused on establishing high‐dimensional flow cytometry for gliomasphere research. In doing so, we measured the full set of possible marker combinations, detected a novel signature and characterised it in silico (e.g. TCGA, DAVID). However, a further functional*in vitro* and *in vivo* characterization of cells harbouring the signature has not been performed yet. Such a characterization was not within the boundaries of this marker combinatorics project but it is the evidently necessary next step (see below).

### 4.3 Strengths of the study

This investigation represents a novel combinatorial analysis of a comprehensive set of nine stemness‐associated molecules. Of all the possible combinations of nine markers, we identified the one combination that was consistently present on all seven models – that are diverse and cover gliomaspheres from different origins. To the best of our knowledge, we are the first to use the viSNE algorithm in the setting of glioblastoma stemness markers. The combination of flow cytometry results and survival data from TCGA links laboratory and clinical research. The marker combinations mapped here will be a valuable starting point for other researchers interested in gliomaspheres.

### 4.4 “Stemness” of the cell systems we used

Are the cells we used really “stem cells”? In our view it is unjustifiable and exaggerated to directly regard gliomaspheres as “stem cells.” Nevertheless, they are a valuable, relevant and heavily used *model* system for stem‐*like* cells that has led to countless important insights. As stemness models, gliomaspheres are typically derived in two main ways: While a number of groups use cells exclusively cultured in sphere‐forming conditions from tumor surgery onwards,^45^, ^46^, ^47^ others rely on gliomaspheres generated from cell lines that were initially kept in classical adherent conditions.^39^, ^48^ We used both sources: NCH421K and NCH644 gliomaspheres are well established and characterised as bona fide stem‐like cells with proven stem‐like properties *in vivo* 35, 36, 49 (continuously cultured as spheres). Gliomaspheres derived from the traditional adherent glioblastoma cell lines U87MG and U251MG have also been extensively studied as stemness model systems –*in vitro* 37, 50 as well as *in vivo*.^41^ Further, we complemented these known models for stemness with gliomaspheres from patients of a clinical trial – cultivated in the same media as the other cells. For these, flow cytometry confirmed the expression of stemness‐related markers but *in vivo* experiments are still missing ‐ which is why we call them “spheres” and never “stem cells” or “stem‐like cells.”

Cells harbouring the CD44+/CD133+/ITGA6+/CD36+ signature were consistently found in gliomasphere cells from all these sources in our study ‐ of which the majority (4/7) were pre‐established stemness models. It is, thus, justifiable to see the identified molecule combination as a potentially dominant consensus signature.

Given that we had deliberately focused on molecules already connected to glioblastoma stemness in the literature, it seems legitimate to regard the CD44+/CD133+/ITGA6+/CD36+ signature as “stemness‐*associated*.” All markers had already individually been described as stemness‐related,^29^, ^30^, ^31^, ^33^ which is why they were chosen. The signature was detectable on all cells, including those with a previously reported “stem‐like” nature *in vivo* (NCH421K, NCH644). Summing up, we deduct that a combination of markers with proven “stemness”‐*properties* measured on proven “stemness”‐*models* can then be also “stemness”‐*related*. Nothing more but also nothing less. In terms of exact data interpretation, we still continuously call the identified marker combination the “CD44+/CD133+/ITGA6+/CD36+ signature” and not the “stemness signature” or anything similar.

### 4.5 Necessity of subsequent functional characterization experiments

The observed fact that overall marker expression is variable across gliomaspheres is notable. On the one hand, given the genetic instability of cancer cells,^51^ this is not a surprising finding. Especially under culture conditions, cells show high phenotypical plasticity. On the other hand, however, the core population was consistently detected and only the additional expression of markers was variable. This indicates that chance and instability are not the only drivers. Rather, the CD44+/CD133+/ITGA6+/CD36+ core population might represent indeed a stable cell phenotype. Additional populations are potentially more chance‐driven. Interestingly, gliomaspheres also resemble the initial tumor more closely than cells under traditional culture conditions.^9^

Given these considerations, it is to be expected that also *in vivo* a subpopulation of cells with the CD44+/CD133+/ITGA6+/CD36+ signature can be found. As mentioned, further research to validate our*in vitro* findings is therefore necessary. On the one hand, it will be helpful to directly confirm the signature in large series of human brain tumor tissue sections – eg via fluorescence in situ hybridization. On the other hand, comprehensive functional experiments are needed: eg it will be interesting to select CD44+/CD133+/ITGA6+/CD36+ cells and to evaluate their tumor formation capacity in NOD‐SCID mice brains. Similarly, it will be important and relevant for a thorough functional characterization of CD44+/CD133+/ITGA6+/CD36+ cells to perform limiting dilution experiments*in vitro*. For these, cells should be sorted based on single markers, different combinations or the identified CD44+/CD133+/ITGA6+/CD36+ signature followed by an assessment of the respective sphere‐forming capacity. An analysis of stem cell markers/signatures (via PCR, immunoblotting, or RNA‐sequencing) from sorted populations would also be useful.

Additionally, it will be interesting to examine the expression of the CD44+/CD133+/ITGA6+/CD36+ signature on neurospheres that represent regular neural stem cells.

Theoretically, it might be that after all these experiments, cells with the CD44+/CD133+/ITGA6+/CD36+ signature can indeed be described as a phenotype especially characteristic of stem‐like glioblastoma cells. Until then, the exact functional status of CD44+/CD133+/ITGA6+/CD36+ cells remains unknown. While the principal goal of introducing high‐dimensional combinatorics analyses to gliomasphere research was successfully achieved in this paper, functional implications are the subject of subsequent studies.

### 4.6 Combination of flow cytometry of surface molecules and TCGA survival data

One important caveat has to be considered when interpreting the connection of the flow cytometry‐identified signature and the TCGA data. Flow cytometry showed that the CD44+/CD133+/ITGA6+/CD36+ combination of markers is present on the surface of single gliomasphere cells. The TCGA analysis, however, could only indicate whether the same (mRNA) marker combination is impactful when abundant in the *full system* of the *whole* glioblastoma tissue. Because the TCGA data is not derived from single cells but rather a transcriptional profiling of *full* cancer tissue ‐ comprising stem‐like cancer cells, bulk cancer cells as well as invading (immune) cells and stroma cells.

Nevertheless, the finding that an enrichment of the signature molecules in the full tissue system is survival‐related is still of interest. It indicates that the signature identified might be indeed connected to a more “aggressive” whole‐system phenotype ‐ eg due to a more stem‐like nature of the tissue. All functional considerations (see above) are, however, still purely speculative at that stage. Future research will have to clarify that aspect ‐ and whether the signature is also present on single glioblastoma cells *in vivo*. For the latter, it will be eg useful to stain tissue sections with multiple markers.

### 4.7 Preliminary implications of in silico findings

Our observation in the DAVID analysis that the pathways with an enrichment of the core signature markers are eg “cell adhesion” or “cell‐matrix adhesion” is much in line with the very nature of gliomaspheres. For the formation of spheres, “cell adhesion” is required. Thus, the signature molecules might be contributing to the spheric geometry that gliomapheres show. And “cell‐matrix adhesion” fits the fact that glioblastoma stem‐like cells are typically found in perivascular niches.^15^

In silico prediction indicated a potential role of PRKC – in line with complementary observations by others: PRKC phosphorylates CD36 and thereby blocks binding of Thrombospondin‐1 in melanoma cells.^52^ It changes phosphorylation patterns of CD44 that result in alterations of cell motility.^53^ Regarding ITGA6, PRKC is necessary for the chemotactic migration of (squamous) carcinoma cells via its ability to mobilize ITGA6B4 from hemidesmosomes.^54^

While the subtype analysis was inconclusive in our view, the IDH1 observations are of specific interest: the CD44+/CD133+/ITGA6+/CD36+ signature seems associated with IDH1 wildtype status – and so a more aggressive phenotype.

To date, however, all these in silico‐based considerations are purely speculative and warrant further investigation.

## 5 CONCLUSION

Summing up, the present study adds a fresh perspective to a well‐studied field. Its mapping of co‐expression combinatorics and its finding of a CD44+/CD133+/ITGA6+/CD36+ molecule signature on gliomaspheres is a relevant step towards a more complete understanding of stemness‐associated molecules in glioblastoma. Feasibility, usefulness and importance of multidimensional approaches are demonstrated in this study – via the contribution of novel experimental evidence. Functional implications of the CD44+/CD133+/ITGA6+/CD36+ signature remain speculative at this stage and warrant further research.

## CONFLICT OF INTEREST

CV, SK, KF and RR were employees of Activartis GmbH at the time of this investigation. FE and AD were formerly (part‐time) employees of Activartis GmbH. The authors declare that the research was conducted in the absence of any other commercial, financial or personal relationships that could be construed as a potential conflict of interest.

## AUTHOR CONTRIBUTION

FE designed and performed experiments, analysed the data (including the viSNE algorithm) and wrote the paper manuscript. BB analysed flow cytometry data and wrote parts of the manuscript. GZ, DP, SK, KF, RR, and AH participated in the experimental work. DL, SS, and WB contributed patient‐derived cell lines. KS, CV and AD planned the project, supervised experiments and edited the manuscript.
