# Supplementary material for: Gliomasphere marker combinatorics: multidimensional flow cytometry detects CD44+/CD133+/ITGA6+/CD36+ signature
Source: J Cell Mol Med. 2018 Nov 22;23(1):281–92. doi: 10.1111/jcmm.13927 (PMC6307809; doi:10.1111/jcmm.13927)
Supplement: Supplementary file 1 [file JCMM-23-281-s001.pdf]

Supplementary Material

Gliomasphere marker combinatorics: multidimensional flow cytometry detects CD44+/CD133+/ITGA6+/CD36+ signature

Friedrich Erhart[1,2,3*], Bernadette Blauensteiner[3], Gabriel Zirkovits[3], Dieter Printz[4], Klara Soukup[3], Simone Klingenbrunner[5], Katrin Fischhuber[5], René Reitermaier[5], Angela Halfmann[3], Daniela Lötsch[6], Sabine Spiegl-Kreinecker[7], Walter Berger[6], Carmen Visus[5], Alexander Dohnal[3]

[1]Department of Neurosurgery, Medical University of Vienna, Vienna, Austria

[2]Institute of Neurology, Medical University of Vienna, Vienna, Austria

[3]Department of Tumor Immunology, St. Anna Kinderkrebsforschung Children's Cancer Research Institute, Vienna, Austria

[4]FACS Core Unit, St. Anna Kinderkrebsforschung Children's Cancer Research Institute, Vienna, Austria

[5]Activartis Biotech GmbH, Vienna, Austria

[6]Institute for Cancer Research, Comprehensive Cancer Center, Medical University of Vienna, Vienna, Austria

[7]University Clinic for Neurosurgery, Kepler University Hospital, Johannes Kepler University, Linz, Austria

*** Correspondence:**

Friedrich Erhart, MD
Department of Neurosurgery, Medical University of Vienna
Währinger Gürtel 18-20, 1090 Vienna, Austria
friedrich@erhart.info, Tel +43/1/40400-45600, Fax +43/1/40400-45660

1 Supplementary Figures

Supplementary Figure 1. Summary of the research process. First, we performed a single-color marker analysis of the 7 different gliomaspheres. This gave us the information what markers are positive in every single of them individually. Based on that information we performed a multi-marker analysis via individual gating or via the viSNE algorithm. Finally, we studied the importance of marker combinations via The Cancer Genome Atlas (TCGA) or the DAVID proteomics database.

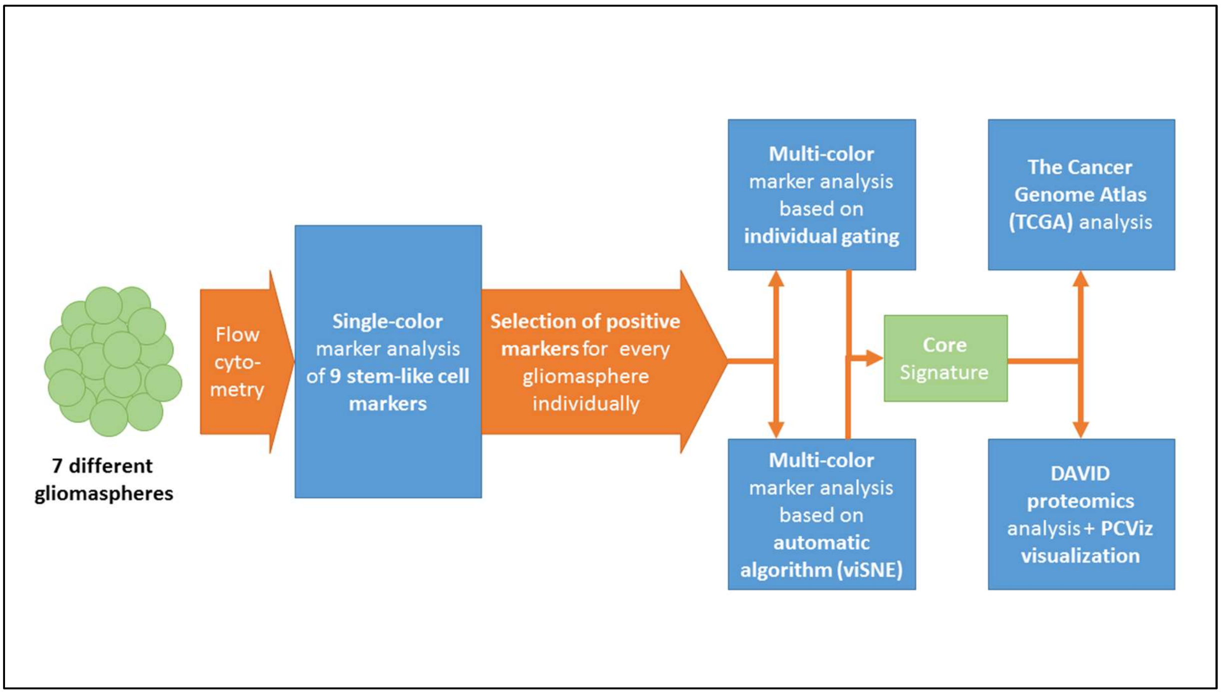

Supplementary Figure 2. Multi-color gating for gliomaspheres of Linz1. Dark-gray curves represent expression levels of target-specific antibodies and light-gray curves isotypes (or for CD36 the unstained control as no isotype was available).

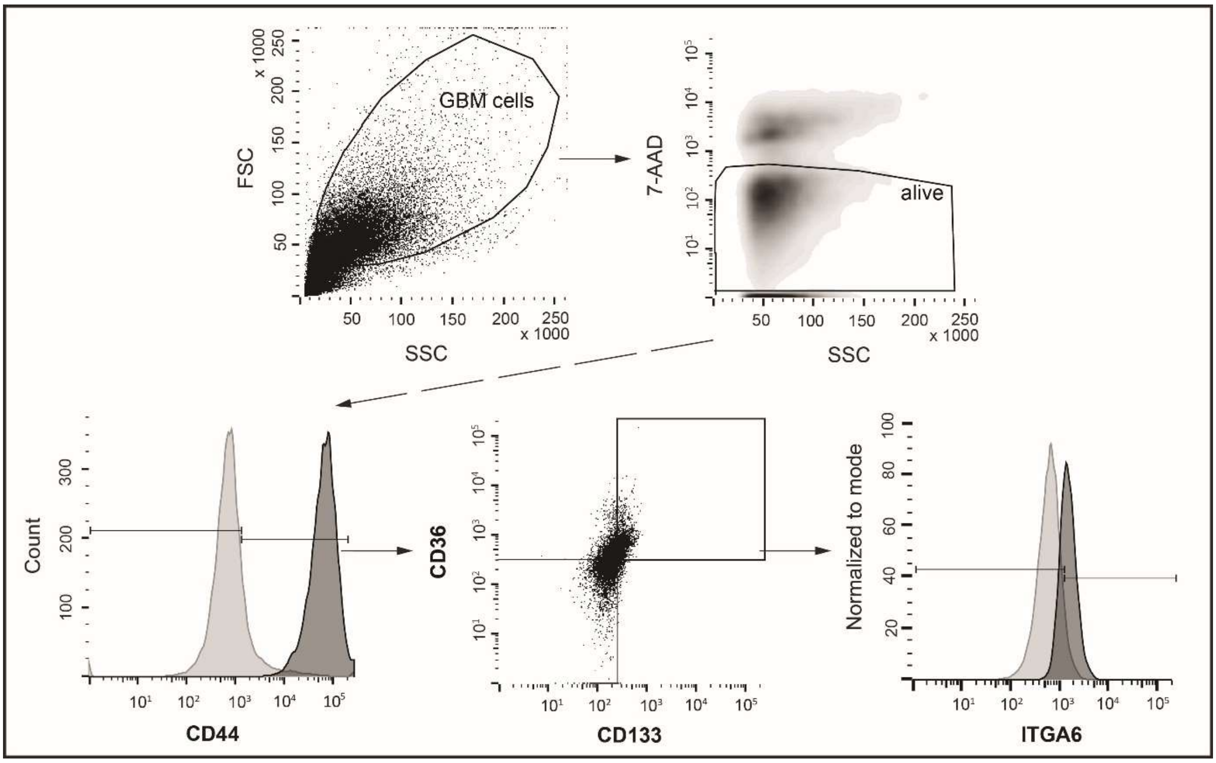

Supplementary Figure 3. Multi-color gating for gliomaspheres of Linz2. Dark-gray curves represent expression levels of target-specific antibodies and light-gray curves isotypes (or for CD36 the unstained control as no isotype was available).

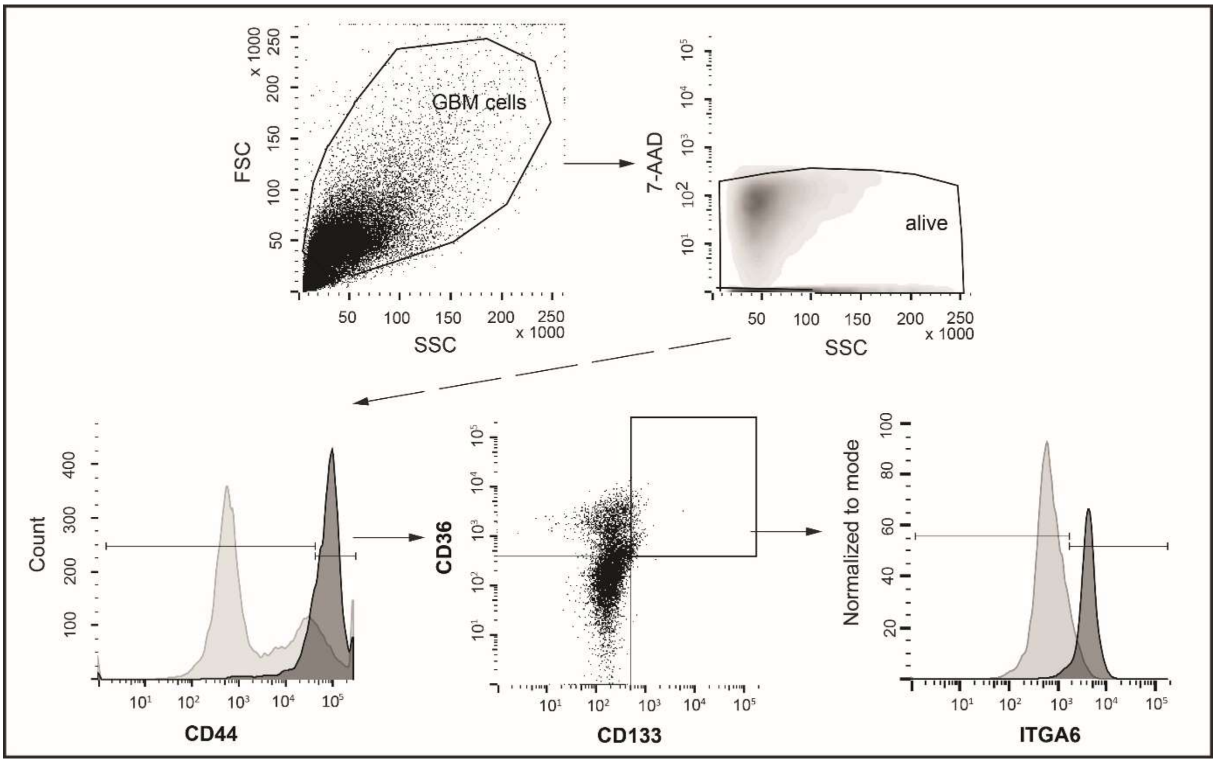

Supplementary Figure 4. Multi-color gating for gliomaspheres of Gli16. Dark-gray curves represent expression levels of target-specific antibodies and light-gray curves isotypes (or for CD36 the unstained control as no isotype was available).

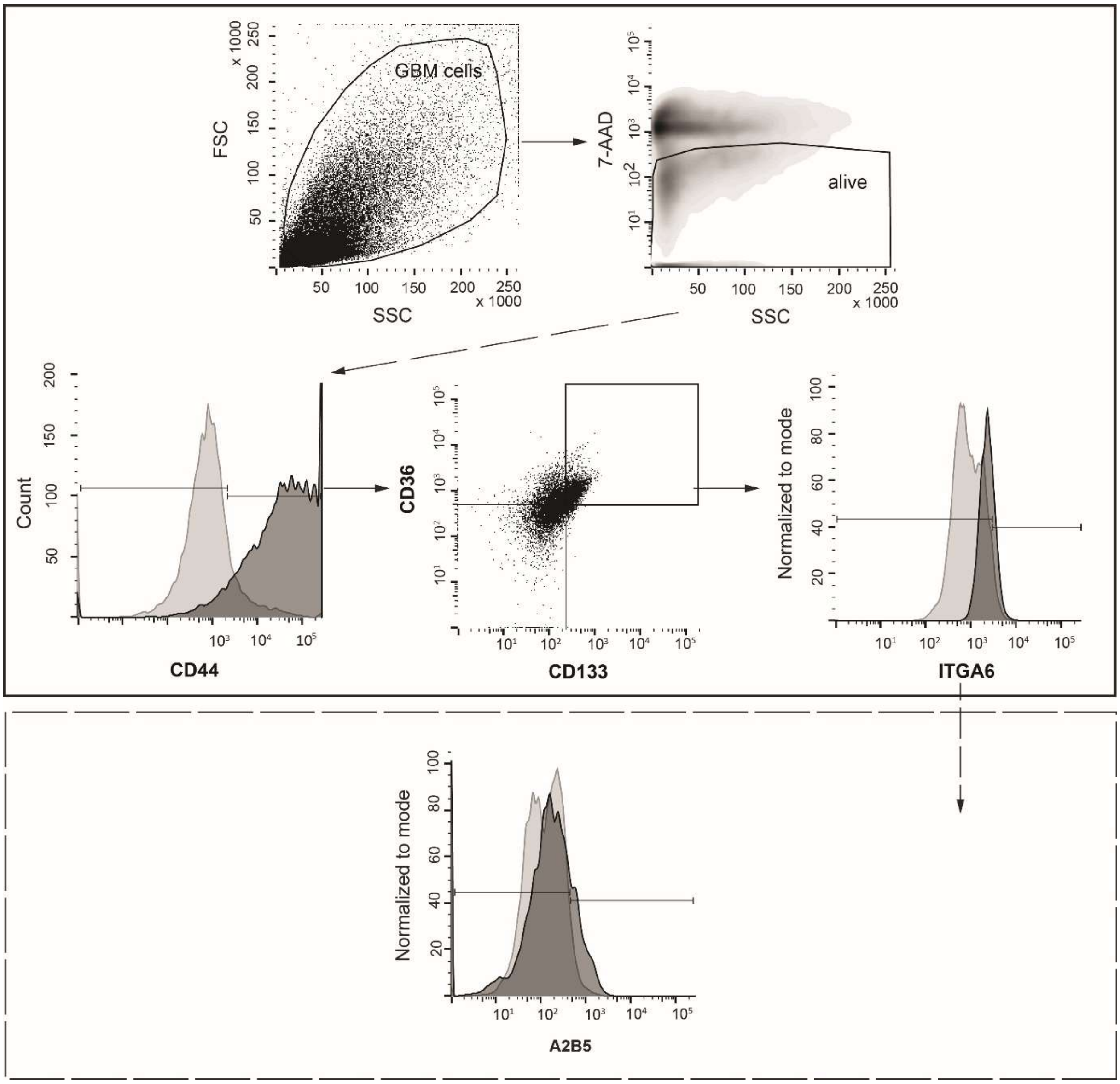

Supplementary Figure 5. Multi-color gating for gliomaspheres of U87MG. Dark-gray curves represent expression levels of target-specific antibodies and light-gray curves isotypes (or for CD36 the unstained control as no isotype was available).

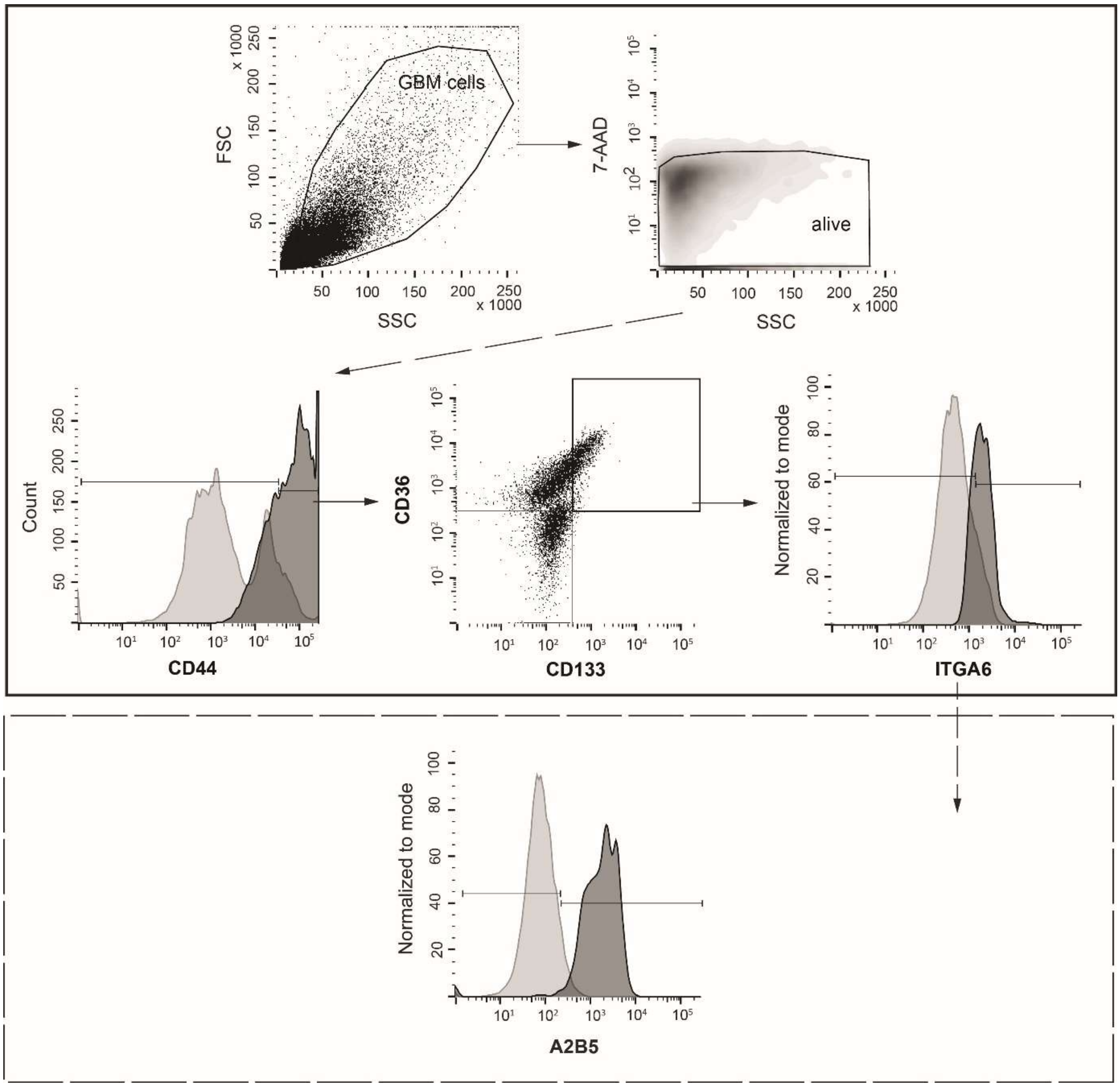

Supplementary Figure 6. Multi-color gating for gliomaspheres of U251MG. Dark-gray curves represent expression levels of target-specific antibodies and light-gray curves isotypes (or for CD36 the unstained control as no isotype was available).

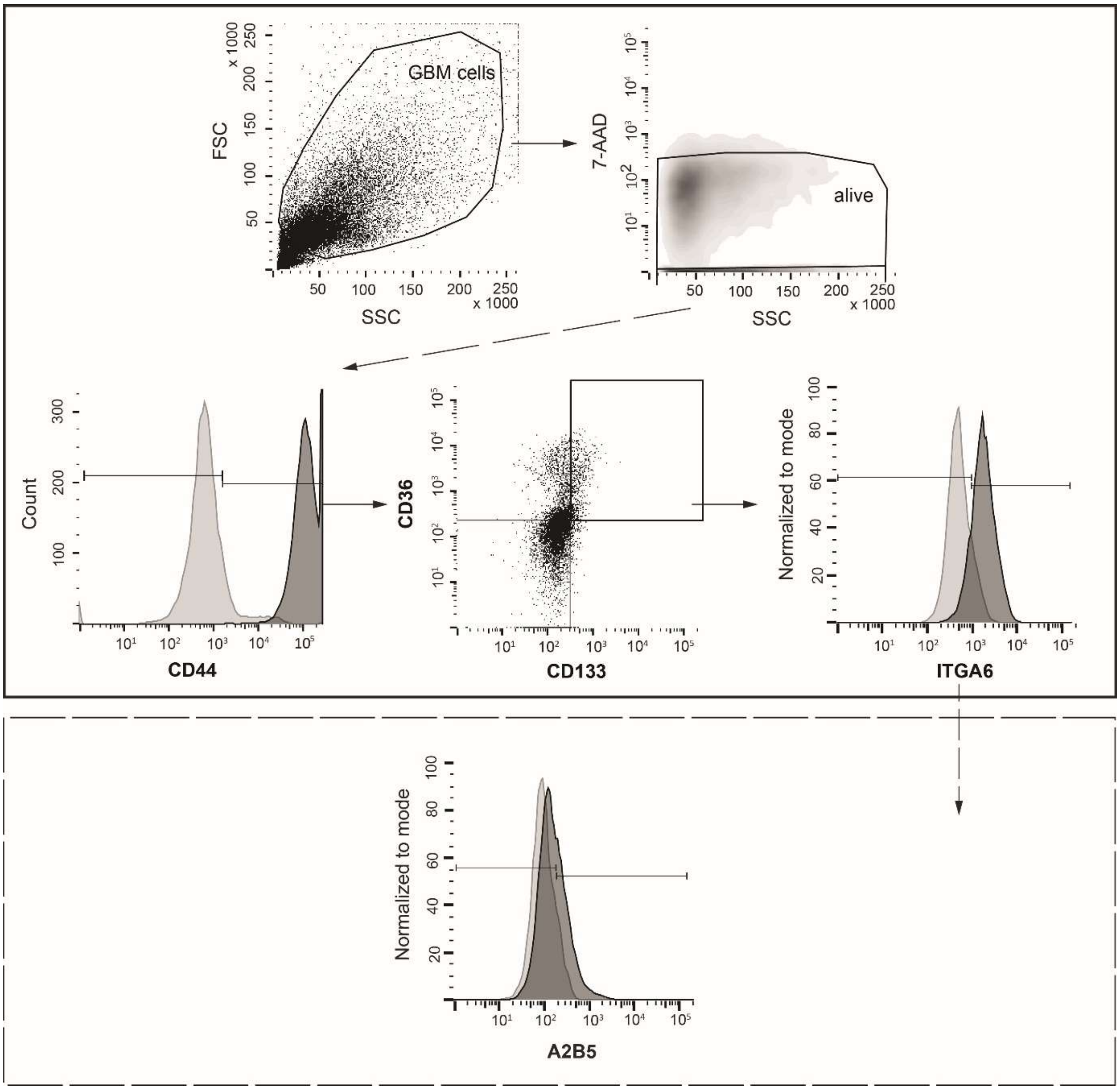

Supplementary Figure 7. Multi-color gating for gliomaspheres of NCH421K. Dark-gray curves represent expression levels of target-specific antibodies and light-gray curves isotypes (or for CD36 the unstained control as no isotype was available).

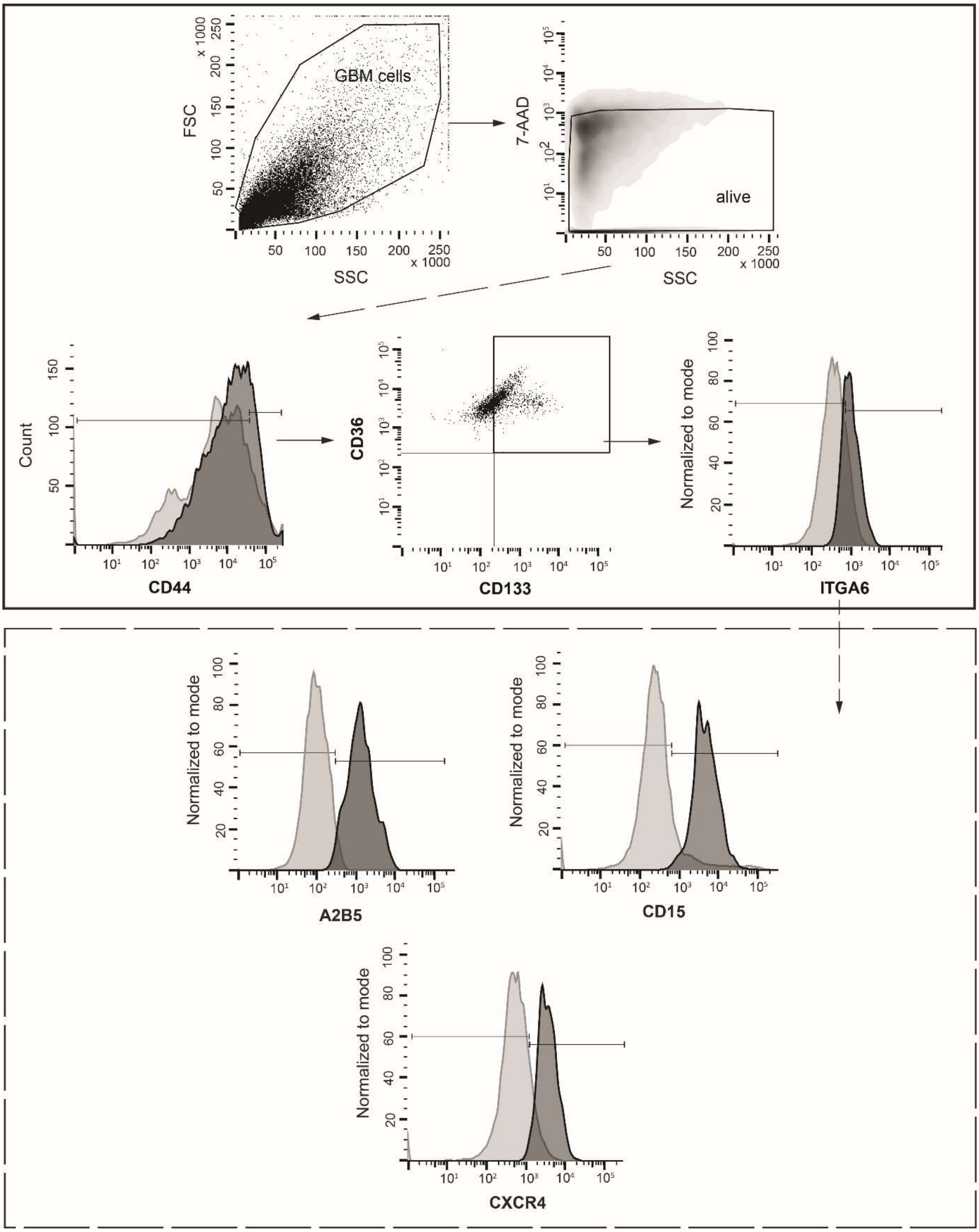

Supplementary Figure 8. Results of the Pathway Commons Network Visualizer (PCViz). Depicted is the outcome if entering the 4 signature genes (CD44/CD36/CD133/ITGA6), focusing on state change control interactions and zooming in on the 200 most relevant genes. While CD36, CD44 and ITGA6 all share the interaction partner Protein Kinase C α (PRKCA), this is not the case for CD133 (PROM).

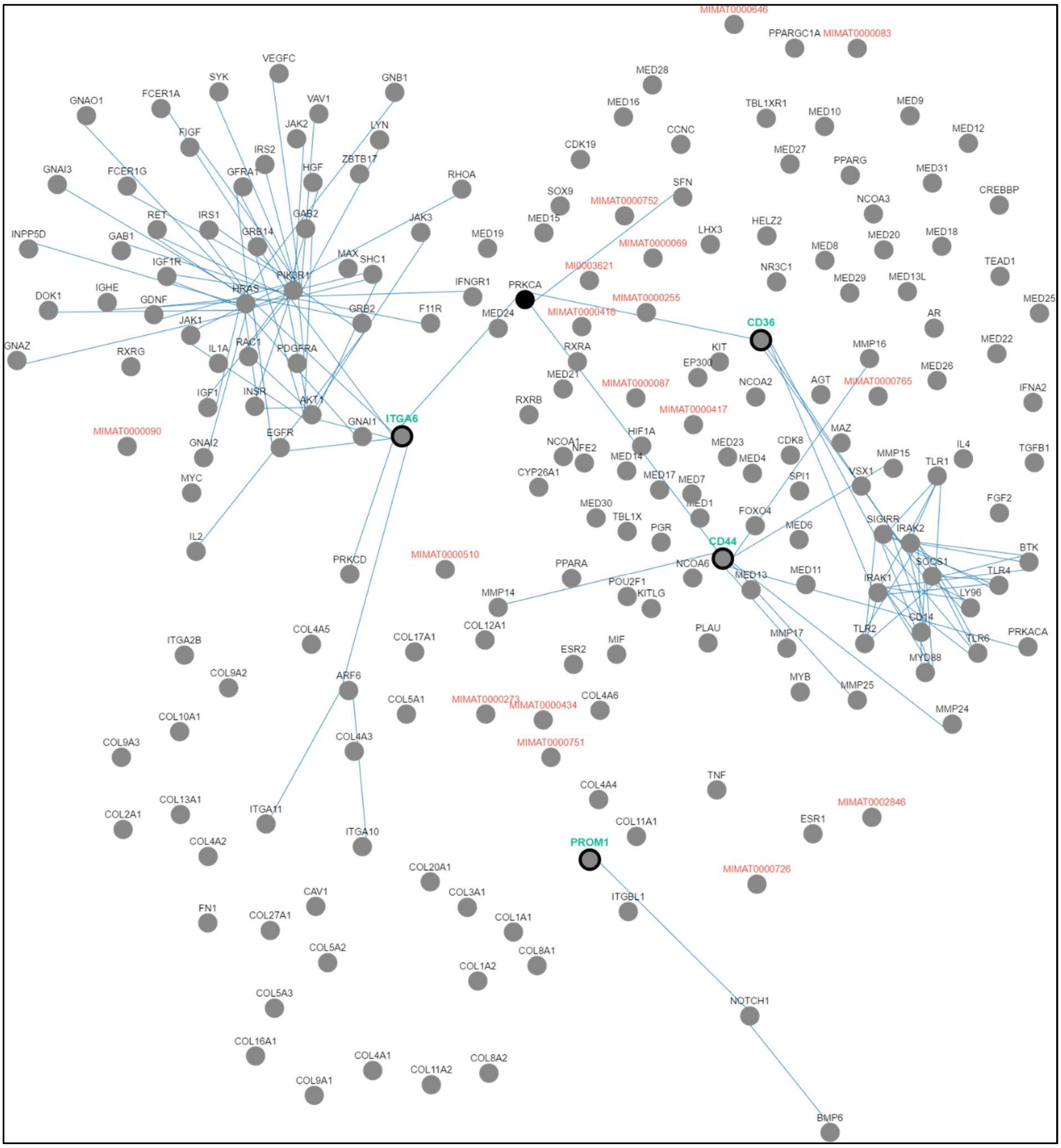

Supplementary Figure 9. Relation of the CD44/CD36/CD133/ITGA6 signature and IDH1 status. Based on TCGA data (MIT/Harvard and North Carolina datasets), the relative intensity of the signature was assessed in the IDH1 mutated and wild-type subtype.

MIT/Harvard dataset

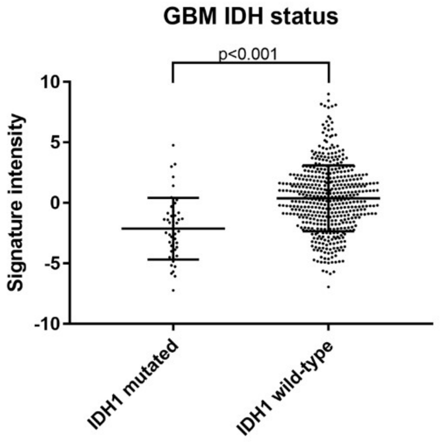

North Carolina dataset

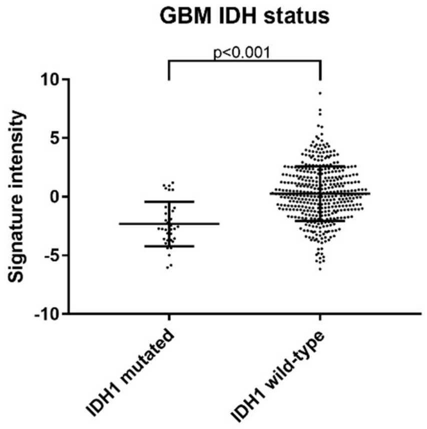

2 Supplementary Tables

Supplementary Table 1. Antibody panel used for the measurement of the 9 stem-like cell markers. Given are the target-specific antibodies used as well as their respective isotypes.

	ANTIBODY	FLUOROCHROME	CLONE	CHANNEL	PRODUCER
1	**CD133/2**	PE	293C3	PE	MACS Miltenyi Biotec
	IgG2b	PE	IS6-11E5-11	PE	MACS Miltenyi Biotec
2	**CD44**	APC-H7	G44-26 / C26	APC-Cy7	BD Pharmingen™
	IgG2b, κ	APC-H7	27-35	APC-Cy7	BD Pharmingen™
3	**CD171 (L1CAM)**	PE-Vio770™	REA163	PE-Cy7	MACS Miltenyi Biotec
	REA control	PE-Vio770™	REA293	PE-Cy7	MACS Miltenyi Biotec
4	**A2B5**	APC	105-HB29	APC	MACS Miltenyi Biotec
	IgM	APC	IS-20C4	APC	MACS Miltenyi Biotec
5	**CD49f (ITGA6)**	eFluor®450	eBioGoH3 (GoH3)	DAPI	eBioscience
	IgG2a, κ	eFluor®450	eBR2a	DAPI	eBioscience
6	**CD15**	BV605	W6D3	Qdot 655	BD Pharmingen™
	IgG1, κ	BV605	X40	Qdot 655	BD Horizon™
7	**CD36**	PerCP-eFluor®710	eBioNL07 (NL07)	488-730/45	eBioscience
	Unstained control*	-	-	-	-
8	**CD184 (CXCR4)**	PE/Dazzle™ 594	12G5	PE-TexasRed	BioLegend
	IgG2a, κ	PE/Dazzle™ 594	MOPC-173	PE-TexasRed	BioLegend

**9**	**IL6 Rα**	FITC	#17506	FITC	R&D Systems
	IgG1	FITC	#11711	FITC	R&D Systems
**10**	**-**	**7AAD**	-	PerCP	eBioscience

* For CD36 there was no isotype control available by the producer eBioscience nor from any other supplier. Therefore, we used unstained cells here as a control.

Supplementary Table 2. Depiction of the MFI values of flow cytometry measurements. All flow cytometry measurements were performed twice in two independent experiments. Both two experiment runs are shown. (A) depicts experiment 1, (B) depicts experiment 2. The MFI values are given in the format “MFI target-specific antibody / MFI isotype”. A difference of at least 10% in the MFI value of target-specific antibodies versus isotypes was considered as “positivity” of the marker.

A Experiment run 1

	**Marker**	A2B5	**CD133**	CD15	**CD36**	**CD44**	CXCR4	IL6R	**ITGA6**	L1CAM
**Cell lines**	Linz1	157/144	184/109	260/512	314/81	64962/736	1320/1117	143/157	1201/875	136/164
	Linz2	161/123	183/152	251/459	171/74	92776/857	1320/963	122/130	2278/919	194/197
	Gli16	166/124	200/96	254/525	463/49	42929/770	1004/1067	121/127	1070/836	136/147
	U87MG	164/101	175/98	142/277	580/33	69253/1464	834/611	14093/13705	492/451	92,7/102
	U251MG	173/107	170/110	183/310	143/36	119828/661	927/867	87/91	2384/663	108/98
	NCH421K	417/96	419/55	401/248	1487/21	14339/6675	763/593	57/73	566/419	164/180
	NCH644	95,9/81	222/61	115/176	132/13	1201/383	506/416	44/50	801/346	198/64

B Experiment run 2

	**Marker**	A2B5	**CD133**	CD15	**CD36**	**CD44**	CXCR4	IL6R	**ITGA6**	L1CAM
**Cell lines**	Linz1	151/141	174/97	312/523	500/93	69253/533	1155/1007	178/180	1370/1151	92/124
	Linz2	180/121	140/99	257/382	752/106	65359/7225	1395/839	165/190	1080/1083	153/316
	Gli16	3414/106	185/111	479/667	2596/174	9918/1753	1194/1609	295/284	2549/2060	214/331
	U87MG	140/130	106/60	128/233	292/63	31464/442	745/849	86/87	596/483	80/79
	U251MG	119/112	86,87/45	216/183	267/61	43059/445	684/720	59/61	1162/457	110/100
	NCH421K	344/127	503/49	1835/241	2636/18	17855/8888	1780/944	56/71	761/492	589/430
	NCH644	149/128	600/62	486/212	2588/9	22643/15759	2768/1505	70/87	1120/453	905/348

Supplementary Table 3. Evaluation of double marker positivity of all studied gliomaspheres. Cells were analyzed for marker double positivity. + = Cells co-expressing the two markers under investigation; empty areas = no co-expression observed

NCH421K	A2B5	CD133	CD15	CD36	CD44	CXCR4	ITGA6	L1CAM
A2B5		+	+	+	+	+	+	+
CD133	+		+	+	+	+	+	+
CD15	+	+		+	+	+	+	+
CD36	+	+	+		+	+	+	+
CD44	+	+	+	+		+	+	+
CXCR4	+	+	+	+	+		+	+
ITGA6	+	+	+	+	+	+		+
L1CAM	+	+	+	+	+	+	+	

NCH644

	A2B5	CD133	CD15	CD36	CD44	CXCR4	ITGA6	L1CAM
A2B5		+	+	+	+	+	+	+
CD133	+		+	+	+	+	+	+
CD15	+	+		+	+	+		+
CD36	+	+	+		+	+	+	+
CD44	+	+	+	+		+	+	+
CXCR4	+	+	+	+	+		+	+
ITGA6	+	+		+	+	+		+
L1CAM	+	+	+	+	+	+	+	

U251MG

	A2B5	CD133	CD36	CD44	ITGA6
A2B5		+	+	+	+
CD133	+		+	+	+
CD36	+	+		+	+
CD44	+	+	+		+
ITGA6	+	+	+	+	

U87MG	A2B5	CD133	CD36	CD44	CXCR4	ITGA6
A2B5		+	+	+	+	+
CD133	+		+	+	+	+
CD36	+	+		+	+	+
CD44	+	+	+		+	+
CXCR4	+	+	+	+		
ITGA6	+	+	+	+		

Gli16	A2B5	CD133	CD36	CD44	ITGA6
A2B5		+	+	+	+
CD133	+		+	+	+
CD36	+	+		+	+
CD44	+	+	+		+
ITGA6	+	+	+	+	

Linz1

	CD133	CD36	CD44	CXCR4	ITGA6
CD133		+	+		+
CD36	+		+	+	+
CD44	+	+		+	+
CXCR4		+	+		
ITGA6	+	+	+		

Linz2

	A2B5	CD133	CD36	CD44	CXCR4	ITGA6
A2B5			+	+	+	
CD133			+	+		+
CD36	+	+		+	+	+
CD44	+	+	+		+	+
CXCR4	+		+	+		
ITGA6		+	+	+		
